# *Polygonum multiflorum* Extract Exerts Antioxidative Effects and Increases Life Span and Stress Resistance in the Model Organism *Caenorhabditis elegans* via DAF-16 and SIR-2.1

**DOI:** 10.3390/plants7030060

**Published:** 2018-07-20

**Authors:** Christina Saier, Christian Büchter, Karoline Koch, Wim Wätjen

**Affiliations:** Institute of Agricultural and Nutritional Sciences, Martin-Luther-University Halle-Wittenberg, Weinbergweg 22, 06120 Halle (Saale), Germany; christina.saier@landw.uni-halle.de (C.S.); Christian.buechter@landw.uni-halle.de (C.B.); karoline.koch@landw.uni-halle.de (K.K.)

**Keywords:** aging, life span-extending effects, insulin-signaling, Nrf2, stress resistance, *Caenorhabditis elegans*, *Polygonum multiflorum* extract

## Abstract

Extracts of the Chinese plant *Polygonum multiflorum* (PME) are used for medicinal purposes as well as food supplement due to anti-aging effects. Despite of the common use of these food supplements, experimental data on physiological effects of PME and its underlying molecular mechanisms in vivo are limited. We used the model organism *Caenorhabditis elegans* to analyze anti-aging-effects of PME in vivo (life span, lipofuscin accumulation, oxidative stress resistance, thermal stress resistance) as well as the molecular signaling pathways involved. The effects of PME were examined in wildtype animals and mutants defective in the sirtuin-homologue SIR-2.1 (VC199) and the FOXO-homologue DAF-16 (CF1038). PME possesses antioxidative effects in vivo and increases oxidative stress resistance of the nematodes. While the accumulation of lipofuscin is only slightly decreased, PME causes a significant elongation (18.6%) of mean life span. DAF-16 is essential for the reduction of thermally induced ROS accumulation, while the resistance against paraquat-induced oxidative stress is dependent on SIR-2.1. For the extension of the life span, both DAF-16 and SIR-2.1 are needed. We demonstrate that PME exerts protective effects in *C. elegans* via modulation of distinct intracellular pathways.

## 1. Introduction

*Polygonum multiflorum* is used in Traditional Chinese Medicine (TCM) and is also very popular as food supplement due to its proposed anti-aging effect [1,2,3]. It has been shown that the stilbene derivative TSG (2,3,5,4′-tetrahydroxystilbene-2-*O*-β-d-glucoside) isolated from this plant is able to increase life span and stress resistance in the model organism *C. elegans* [4]. However, experimental data of protective effects of commercially available *Polygonum multiflorum* extract (PME) in vivo on aging and life span are limited. We investigated effects of PME (up to 1000 µg/mL) on life span, thermal and oxidative stress resistance, the modulation of reactive oxygen species (ROS) production in *C. elegans* as well as the impact of the protein deacetylase SIR-2.1 and the transcription factors DAF-16 and SKN-1 on the outcome of the aforementioned parameters.

Aging is defined as an accumulation of deleterious changes in organelles, cells and tissues with increasing age. The accumulation of these changes is thought to be responsible for the risk of several diseases and finally aging-related death [5]. Oxidative stress is believed to play a role in both physiological aging processes, e.g., modulation of distinct cellular pathways like histone modifications, as well as pathological aging processes, e.g., age-related neurodegenerative diseases [6].

PME possess anti-aging effects in different species: Chan et al. reported that mice fed with PME had less lipofuscin, a species-independent biomarker of aging, in the hippocampus [7]. Furthermore lower concentrations of malondialdehyde (final product of lipid oxidation) were detected in the brains of these animals [7]. An extract consisting of *Polygonum multiflorum* reduced the lipofuscin content in liver and brain tissues in mice [8]. In addition, diverse protective effects on neurodegeneration have been found: Li et al. showed neuroprotective effects of an extract against nigrostriatal degeneration in mice [9]. PME was able to modulate mechanisms associated with the development of Alzheimer’s disease: The accumulation of beta amyloid (Aβ was reduced by modulating APP (amyloid precursor protein) processing in vitro [10] and to prevent Aβ-induced increase of thiobarbituric acid reactive substances and cognitive deficits in mice [11]. Steele et al. reported cytoprotective effects of PME in astroglia cells [12]. Furthermore, an improvement of cognitive performance in senescence accelerated mice [13] and an attenuation of glutamate-induced neurotoxicity [14] was demonstrated after application of PME. Besides these indirect antiaging effects, a direct prolongation of life span by PME has not been reported yet.

However, the stilbene glucoside TSG, a main bioactive component of *Polygonum multiflorum* [15], is able to increase life span in the model organism *C. elegans.* TSG increases the mean life span by 23.5% independent of DAF-16, a central component of the insulin-like signaling pathway [4]. Other compounds of PME are for example physcion, apigenin, hyperoside, rutin, vitexin, beta-amyrin, beta-sitosterol and daucosterol [15]. To connect previous experiments of the isolated component TSG to effects of PME itself, we evaluated the effects of PME on oxidative and thermal stress resistance and lifespan in the model organism *C. elegans* using different strains defective in distinct aging-associated cellular pathways (DAF-16, SIR-2.1).

## 2. Results

### 2.1. PME Increases Life Span, but Not Resistance AGAINST Thermal Stress

**Figure 1:**
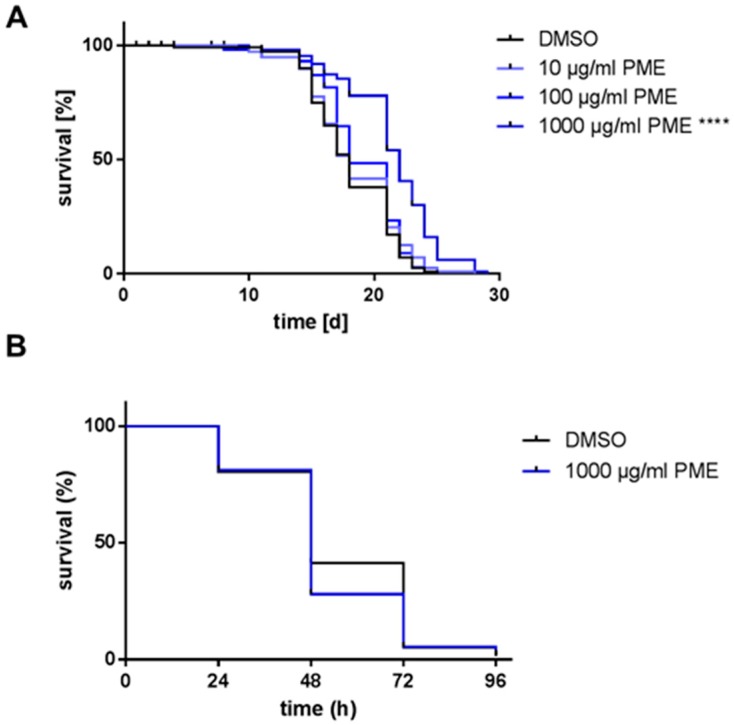
*Polygonum multiflorum* extract (PME) increases life span, but not thermal stress resistance. (**A**) Wild type L4 larvae (N2) were incubated at 25 °C with PME or DMSO (vehicle, 0.4%) and the survival was analyzed by touch-provoked movement. Kaplan-Meier statistics were used for comparison of the survival curves, *n* = 3 (40 individuals per group), Log-Rank (Mantel-Cox)-test, **** *p* < 0.0001. (**B**) Wild type L4 larvae (N2) were treated with 1000 µg/mL PME or DMSO (vehicle, 0.4%) at 20 °C. After 72 h, thermal stress (37 °C) was applied for 3 h. The survival of the nematodes was monitored by touch-provoked movement every 24 h. Kaplan-Meier statistics were used for the comparison of the survival curves, *n* = 3 (60 individuals per group), Gehan–Breslow–Wilcoxon test.

Since PME is traditionally used as anti-aging medicine, we investigated if the extract is able to prolong the life span of the model organism *Caenorhabditis elegans*. Incubation with the highest concentration of the extract (1000 µg/mL) increased the mean life span by 18.6% (21.5 days in PME-treated animals vs. 18.1 days in control animals). The other concentrations used (10, 100 µg/mL) were not able to cause a significant prolongation of the life span (increase of 1.5% and 4.8%, respectively, Figure 1A, Table 1).

This increase in life span caused by PME was not due to the unspecific effect of caloric restriction by reduced food intake, since the rate of pharyngeal pumping was not changed (Figure S1A). We further checked, if PME changes the rate of progeny which may influence the life span, but this was not the case (Figure S1B). We used the highest concentration of PME (1000 µg/mL) to investigate, if the resistance against thermal stress is increased by the extract. However, no protective effect was detectable. In contrary, the resistance against thermal stress was even (non-significantly) decreased by the extract (Figure 1B).

### 2.2. Antioxidative and Radical-Scavenging Effects of PME

**Figure 2:**
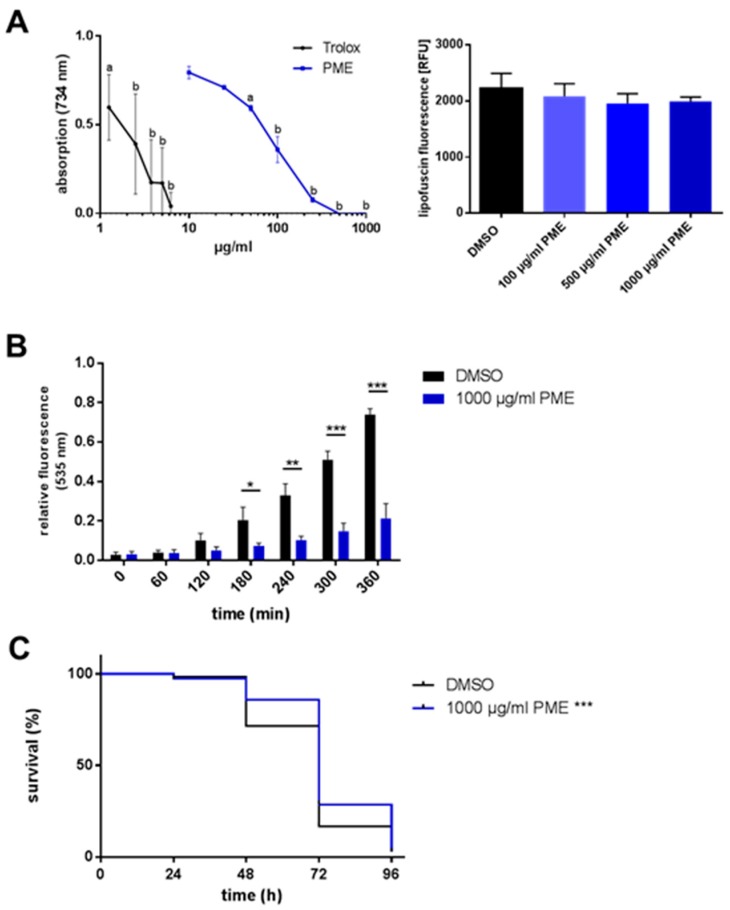
Antioxidative and radical-scavenging effects of PME. (**A**) Radical scavenging assay: In the cell-free TEAC assay, PME exerts antioxidative properties. Values are mean ± SD, *n* = 3, one-way ANOVA with Dunnett’s multiple comparisons test vs. control (0 µg/mL), a: *p* ≤ 0.05, b: *p* ≤ 0.0001 (left side). Lipofuscin accumulation: Wild type L4 larvae (N2) were treated at 20 °C with PME or DMSO (vehicle, 0.4%) for 72 h, followed by 24 h in media without PME, then lipofuscin fluorescence was measured. Values are mean ± SD, *n* = 3 (20 individuals per group), one-way ANOVA with Dunnett’s multiple comparisons test, right side). (**B**) Wild type L4 larvae (N2) were treated with PME or DMSO (vehicle, 0.4%) for 48 h at 20 °C, then transferred into wells of a microtiter plate containing H_2_DCF-DA. The thermally-induced increase in ROS was detected via DCF fluorescence (emission: 535 nm). Values are mean ± SD, normalized to DMSO at 420 min, *n* = 3 (16 individuals per group), unpaired *t*-test, * *p* ≤ 0.05, ** *p* ≤ 0.01, *** *p* ≤ 0.001 vs. DMSO. (**C**) Wild type L4 larvae (N2) were treated with PME or DMSO (vehicle, 0.4%) for 72 h, then nematodes were transferred into PME-free medium containing 50 mm paraquat. Survival of the nematodes was analyzed by touch-provoked movement every 24 h. Kaplan-Meier statistics were used for the comparison of the survival curves, *n* = 3 (60 individuals per group), Gehan–Breslow–Wilcoxon test, *** *p* = 0.0004.

PME is thought to exert potent antioxidative effects due to its high content in flavonoids and stilbenes. This effect was shown in many studies in vitro. We used the TEAC (trolox equivalent antioxidative capacity) assay as a cell-free assay system to estimate the radical-scavenging effects of PME in comparison to the vitamin E derivative trolox, a well-known radical scavenger. We clearly see a radical scavenging effect of PME (first significant effects at 50 µg/mL), but the vitamin E derivative is much more effective in neutralizing the colored radical (Figure 2A, left side). Next, we analyzed the antioxidative properties of PME in vivo by investigating the content of the aging marker lipofuscin in *C. elegans*. Fluorescent lipofuscin granules consist of highly oxidized proteins and lipids and are taken as marker for oxidative stress. However, treatment with PME (100, 500, 1000 µg/mL) resulted only in a small, but not significant reduction of lipofuscin fluorescence (Figure 2A, right side). In contrast to the results shown for lipofuscin, 1000 µg/mL PME strongly protects against the thermally-induced accumulation of reactive oxygen species: Compared to the relative fluorescence units of the control value (rfu: 0.74 ± 0.017), the PME-treated nematodes exhibit a strongly reduced DCF fluorescence value of 0.21 ± 0.04 rfu (at 6 h). This strong effect is not due to quenching phenomena of the extract (see Supplementary Figure S1C) or reduced body size of the nematodes (see Figure S1D). Using lower concentrations of PME (1–500 µg/mL), no significant decrease in DCF fluorescence was detectable (Figure S2A). The antioxidative effect of PME was also traceable as a protection against paraquat, a chemical which generates oxidative stress via redox-cycling [16,17]. Incubation of *C. elegans* with paraquat (50 mm) represents a lethal stress so that the nematodes start to die 24 h after application of the stress. Pre-treatment with PME (1000 µg/mL) resulted in an increased resistance against paraquat-induced oxidative stress (Figure 2C, Table 2). Using lower concentrations of PME (1–500 µg/mL), only 500 µg/mL was able to exert a significant protective effect (Figure S2B).

### 2.3. PME Modulates the Intracellular Localization of DAF-16, but Not SKN-1

**Figure 3:**
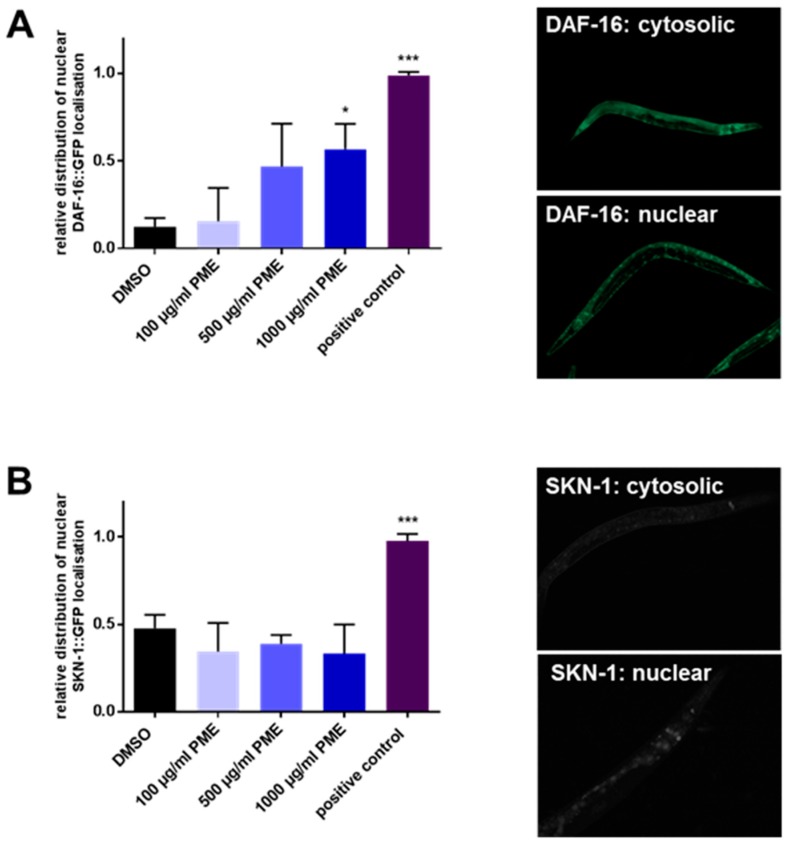
PME induces nuclear translocation of DAF-16, but not of SKN-1 in *C. elegans*. (**A**) DAF-16 localization was analyzed via fluorescence microscopy, using the transgenic strain TJ356 (DAF-16::GFP). The nematodes were incubated for 1 h at 20 °C with PME or DMSO (vehicle, 0.4%). The nematodes were classified in animals showing cytosolic or nuclear GFP fluorescence. Values are mean ± SD, *n* = 3 (30 individuals per group), one-way ANOVA with Dunnett’s multiple comparisons test, * *p* ≤ 0.05; *** *p* ≤ 0.001. Positive control: thermal stress (37 °C, 5 min); (**B**) SKN-1 localization was analyzed via fluorescence microscopy using the transgenic strain LD001 (SKN-1::GFP). The nematodes were incubated for 1 h at 20 °C with PME or DMSO (vehicle, 0.4%). The nematodes were classified in animals showing cytosolic or nuclear GFP fluorescence. Values are mean ± SD, *n* = 3 (30 individuals per group), one-way ANOVA with Dunnett’s multiple comparisons test, *** *p* ≤ 0.001. Positive control: 4 mm H_2_O_2_.

We analyzed if the insulin-like signaling (IlS) pathway is involved in the protective effects caused by PME. The IlS pathway is a pivotal aging and stress response associated pathway that regulates the activities of the transcription factors DAF-16 and SKN-1. Using transgenic DAF-16: GFP and SKN-1: GFP nematodes the localization of the transcription factors was investigated. The nuclear localization of the transcription factor DAF-16 is strongly enhanced by PME: 1000 µg/mL PME increases the amount of nematodes with a mainly nuclear phenotype 4.65-fold (control: 12.2% vs. 56.7% PME 1000 µg/mL). 500 µg/mL caused a similar, but non-significant effect, while 100 µg/mL caused no increase in the amount of animals with nuclear localized DAF-16 (Figure 3A). Next, we analyzed the localization of SKN-1, a homologue of the mammalian Nrf2. However, no increase in the nuclear localization of SKN-1 was detectable, even at the highest concentration used (Figure 3B).

### 2.4. Effects of PME in C. elegans Are Partly Dependent on DAF-16 and SIR-2.1

**Figure 4:**
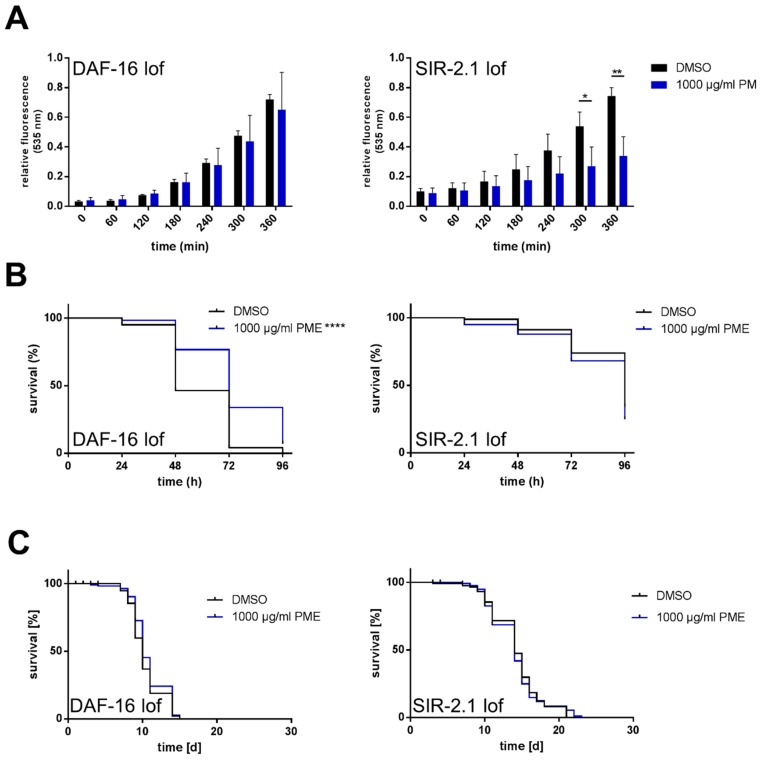
Effects of PME in *C. elegans* are partly dependent on DAF-16 and SIR-2.1: (**A**) Antioxidative potential: L4 larvae of the DAF-16-deficient strain CF1038 (left side) or the SIR-2.1-deficient strain VC199 (right side) were treated with PME or DMSO (vehicle, 0.4%) for 48 h before they were transferred into wells containing H_2_DCF-DA. Thermally-induced increase in ROS was detected via fluorescence intensity of DCF (535 nm). Values are mean ± SD, normalized to DMSO at 420 min, *n* = 3 (16 individuals per group), unpaired *t*-test, * *p* = 0.0446, ** *p* = 0.0079. (**B**) Resistance to paraquat stress: L4 larvae of the DAF-16-deficient strain CF1038 (left side) or the SIR-2.1-deficient strain VC199 (right side) were treated with PME or DMSO (vehicle, 0.4%) for 72 h and subsequently transferred into PME-free medium containing 50 mm paraquat. Survival of the nematodes was monitored by touch-provoked movement. Kaplan-Meier statistics were used for the comparison of the survival curves, *n* = 3 (60 individuals per group), Gehan–Breslow–Wilcoxon test (left side: **** *p* < 0.0001, right side: no significant difference between groups). (**C**) Analysis of life span: L4 larvae of the DAF-16-deficient strain CF1038 (left side) or the SIR-2.1-deficient strain VC199 (right side) were incubated at 25 °C with PME or DMSO (vehicle, 0.4%) and the survival of the nematodes was analyzed by touch-provoked movement five times a week. Kaplan–Meier statistics were used for the comparison of the survival curves, *n* = 3 (40 individuals per group), Log-Rank (Mantel-Cox)-test (no significant difference between groups).

We used DAF-16-deficient nematodes to investigate if the antioxidative and life-prolonging effects of PME depend on functional DAF-16. Repeating the experiment from Figure 2B with DAF-16-deficient nematodes, no reduction of DCF fluorescence was detectable (non-significant reduction of 9.5% after 6 h of thermal stress), demonstrating a requirement of DAF-16 for the reduction of ROS accumulation by PME (Figure 4A, left side). However, the protective effects of PME against paraquat-induced lethal oxidative stress was independent of this factor: Performing the experiment from Figure 2C with DAF-16-deficient nematodes, still a strong increase in survival of the nematodes was detectable (Figure 4B, left side): PME (1000 µg/mL) resulted in a 25.8% increase in nematode survival from 58.9 h to 74.1 h. Repeating the life span experiment (Figure 1A) with DAF-16-deficient nematodes, no significant prolongation of life span was detectable (life span of DMSO treated animals: 10.36 days, PME (100 µg/mL)-treated nematodes: 10.74 days) showing a requirement of DAF-16 for life span prolongation caused by PME.

Sirtuins, proteins that show e.g., histone deacetylase activity, play a pivotal role in the molecular mechanism of life prolongation. Therefore, we used a SIR-2.1-deficient nematode strain (VC199) to analyze if this protein is necessary to exert the effects of PME. Since the radical scavenging effect of PME in this strain is comparable to that in wild-type animals (Figure 4A), SIR-2.1 seems not to be responsible for that protective effect of PME. On the other hand, using this SIR-2.1-deficient strain, PME was not able to protect against paraquat stress (Figure 4B, right side: mean survival of DMSO-treated nematodes: 87.2 h, mean survival of PME (1000 µg/mL)-treated nematodes: 84.21 h) and caused no increase in life prolongation (Figure 4C, right side: mean life span of DMSO-treated nematodes: 14.16 days, mean life span of PME (1000 µg/mL)-treated nematodes: 14.05 days). Therefore, we conclude that SIR-2.1 is necessary for the enhancement of life span by PME as well as the increased stress resistance against paraquat.

## 3. Discussion

We analyzed aging-related effects of PME in the model organism *Caenorhabditis elegans*. PME is widely used as anti-aging agent due to its phytochemicals, e.g., flavonoids and stilbene derivatives. However, life prolonging effects of PME were not demonstrated before and experiments performed with important phytochemicals of the plant are limited. The stilbene derivative TSG (2,3,5,4′-tetrahydroxystilbene-2-*O*-β-d-glucoside) as a major component of PME was already investigated in the model organism *C. elegans:* The compound exerted a high antioxidative capacity both in a cell-free assay and in the nematode. TSG increased the resistance of *C. elegans* against lethal thermal stress more prominently than resveratrol. The antioxidative capacity of TSG was even higher compared to resveratrol. The level of the aging pigment lipofuscin was decreased after incubation with TSG and the stilbene derivative extends the mean, median and maximum adult lifespan of *C. elegans* [4].

We were able to show a life-prolonging effect of the *Polygonum multiflorum* extract (18.6% elongation of mean life span; 1000 µg/mL PME) consisting of different phytochemicals. According to the results of life span analysis it is clear that PME modulates the DAF-16 as well as the SIR-2.1 pathway in *C. elegans*: While PME (1000 µg/mL) increases the mean life span in the wild type nematodes by 18%, no significant effect is detectable using the same concentration in mutants defective in DAF-16 (+3%) and SIR-2.1 (−0.7%). Furthermore, the modulation of DAF-16 is more relevant for the PME-mediated reduction of ROS during thermal stress, while the modulation of SIR-2.1 on the other hand is more relevant for the protective effect of the extract against stress induced by the redox-cycler paraquat. Both results show clearly that the effects of PME are not just mediated via unspecific radical scavenging effects of components of the extract (as shown in the TEAC assay), but require a specific interaction with distinct signaling pathways of the nematode.

Studies on the longevity promoting effect of resveratrol, a minor compound of PME, have been partly inconclusive which is to some extent due to different experimental conditions (different strains, different stages): Upadhyay et al. [18] reported an increase of life span after treatment with resveratrol (100 µm). Zarse et al. [19] reported that resveratrol significantly extends *C. elegans* lifespan already at a concentration of 5 µm by 3.6% (mean lifespan) and 3.4% (maximum lifespan). On the other hand, Chen et al. [20] observed no extension of the normal life span of *C. elegans* either in liquid or solid growth media containing different concentrations of resveratrol.

TSG-mediated extension of lifespan was not abolished in a daf-16 loss-of-function mutant strain [4] showing that this aging-related transcription factor is not involved in the effects of TSG. On the other hand, our new data on PME show that the prolongation of life span is totally abolished using a DAF-16-defective mutant. This shows that TSG may be a prominent component of PME, but the life-prolonging effects of the extract are also dependent on other components. Since the dependence on the pivotal insulin-like signaling pathway is different between PME and TSG, multiple components in the extract are likely to interact together, so the effect of the component TSG alone is not visible. Bass et al. [21] analyzed effects of resveratrol in *C. elegans* (wild type and sir-2.1 mutant nematodes) but their results were variable: Resveratrol treatment results in slight increases in lifespan in some trials but not others (wild type and sir-2.1 mutant animals). As an explanation for the different effects there may be variations from one study to another concerning the delivery of the compounds to the nematodes. The use of liquid or solid growth media containing different concentrations of resveratrol makes it also difficult to compare results between studies.

*Polygonum multiflorum* extract possesses antioxidant effects in vivo, increases resistance against oxidative stress and prolongs the mean life span in the model organism *Caenorhabditis elegans*. Both DAF-16 and SIR-2.1 are required for the extension of the life span. Furthermore, DAF-16 is essential for the reduction of thermal-induced ROS accumulation, while the resistance against paraquat stress is dependent on SIR-2.1. We were able to demonstrate for the first time, that PME exerts protective effects in vivo via modulation of distinct intracellular pathways.

## 4. Materials and Methods

Chemicals were of analytical grade and were purchased from Sigma (Deisenhofen, Germany). *Polygonum multiflorum* aqueous extract powder was obtained from HerbaSinica Hilsdorf GmbH (Rednitzhembach, Germany) and a stock solution in DMSO (250 mg/mL) was prepared for all experiments.

*C. elegans* strains and maintenance: *C. elegans* strains used in this study (wild-type N2 var. Bristol, CF1038 [daf-16(mu86) I.], TJ356 [zIs356 IV (pdaf-16-daf-16: gfp; rol-6)], VC199 sir-2.1 (ok434) IV and LD001 [ldIs007 pskn-1: skn-1b/c: gfp; rol-6]) and bacterial strains were provided by the *Caenorhabditis* Genetics Center (CGC). Strain maintenance was performed at 20 °C on nematode growth medium (NGM) agar plates containing a lawn of *E. coli* var. OP50. Treatment of *C. elegans* with PME/DMSO was conducted in liquid NGM.

Stress resistance: (a) Resistance against oxidative stress (stressor: paraquat): Nematodes were synchronized, and 60 L4 larvae and young adults were treated for three days with different PME concentrations and FUDR (5-fluorodeoxyuridine) to prevent progeny from hatching, then transferred into PME-free medium containing the redox-cycler paraquat (50 mm). In the following four days survival of the nematodes was measured by touch-provoked movement every 24 h. For these experiments the wild-type strain N2 and the loss-of-function mutants CF1038 and VC199 were used. (b) Resistance against thermal stress: Synchronized wild-type L4 larvae and young adults were treated with PME and FUDR for three days, washed in PBST (phosphate buffered saline containing 0.1% Tween 20) and transferred into a 96-well microtiter plate. 60 nematodes per group were then exposed to thermal stress (37 °C) for three hours and transferred into media without PME. Every 24 h the survival of the nematodes was measured by touch-provoked movement for four days.

Measurement of ROS accumulation (DCF assay): Synchronized L4 larvae and young adults were treated with different PME concentrations for 48 h, washed in PBST and transferred into a 384-well microtiter plate. After transfer of the nematodes, H_2_DCF-DA-solution was added to each well to reach a final concentration of 50 µm. During thermal stress (37 °C), fluorescence intensities (excitation: 485 nm; emission: 535 nm) were recorded. Fluorescence values were normalized to the increase of the control value (rfu at t = 7 h). This experiment was performed with the wild-type strain (N2) as well as the loss-of-function mutants CF1038 and VC199. To exclude that PME interferes with the fluorescence of the fluorescent probe e.g., by quenching phenomena, the fluorescence intensity (535 nm) of oxidized DCF (dichlorofluorescein; Sigma) in M9 was analyzed in the presence of different concentrations of PME, as well as the corresponding amount of DMSO using a monochromator-based microplate reader (Synergy Mx; BioTek, black 96 well plates from Greiner, Kremsmünster, Austria).

TEAC assay: Radical ABTS solution (2,2′-Azino-bis(3-ethylbenzothiazoline-6-sulfonic acid)) consisted of 14 mm ABTS and 4.9 mm APS (ammonium peroxodisulfate; 1:1) and was diluted with ethanol (70%) until its absorption at 734 nm measured 1.8. Decoloration of the radical solution was measured in vitro in presence of different concentrations of PME in comparison to different concentrations of the vitamin E derivative trolox at 734 nm.

Lipofuscin detection: After incubation of synchronized L4 wild type larvae and young adults with different concentrations of PME for 72 h, the nematodes were treated without PME for 24 h, transferred on a microscope slide and levamisole was applied to anesthetize the nematodes. 30 Nematodes per group were photographed (excitation: 390/18 nm, emission: 460/60 nm) and the accumulation of fluorescent lipofuscin was analyzed densitometrically (ImageJ, National Institutes of Health, Bethesda, MD, USA).

Intracellular localization of DAF-16: GFP and SKN-1: GFP: The transgenic strains TJ356 and LD001 were used to detect the intracellular localization of the GFP-tagged transcription factors. Synchronized L4 larvae and young adult animals of the corresponding strains were transferred into liquid treatment media and maintained for one hour at 20 °C, respectively. A drop of medium containing the nematodes was placed on a microscope slide, mixed with levamisole and the cellular localization of DAF-16: GFP/SKN-1: GFP was detected. A nematode was categorized as “nuclear” for the corresponding transcription factor, if at least 3 clear and bright nuclei in different areas of the nematode were visible; all other animals are categorized as “cytosolic”.

Life span: For the analysis of the life span at 25 °C the wild-type strain N2 and the loss-of-function mutants CF1038 and VC199 were used. 40 synchronized L4 larvae and young adult animals were transferred into liquid media (day 0 of the life span). During the first 10 days, medium contained 120 µm FUDR to prevent the hatching of viable progeny. The media were exchanged five times a week and the survival of the animals was measured by touch-provoked movement.

Pharyngeal pumping assay: Wild type L4 larvae and young adult animals (N2) were treated for 96 h with PME or DMSO (vehicle, 0.4%) at 20 °C, then nematodes were transferred onto agar-plates. Pharyngeal pumping activity was monitored for 15 s three times per nematode.

Determination of body size and offspring production: Wild type L4 larvae and young adult animals (N2) were treated at 20 °C with PME or DMSO (vehicle, 0.4%) for 72 h, then transferred into media without PME for 24 h. Images of individual nematodes were taken (Olympus BX43) and the body size was determined by measuring the area of each worm (ImageJ, 20 individuals per group). Effect of PME on offspring production was determined by incubation of wild type L4 larvae and young adult animals with PME or DMSO (vehicle, 0.4%) for six days. Each day the nematodes were transferred into new media and progeny was counted (10 individuals per group).

Statistics: Statistical significance was determined by Student’s *t*-test or one-way ANOVA with Dunnett’s post-test while life span analyses, thermal and oxidative resistance was calculated using Kaplan-Meier survival analysis with log-rank (Mantel-Cox) or Gehan–Breslow–Wilcoxon test (PASW Statistics for Windows, SPSS Inc., Chicago, IL, USA; GraphPad Prism 6, La Jolla, CA, USA).

## Figures and Tables

**Table 1:** Data of life span experiments.

Genotype	Treatment	Mean [d] ± SD	% Difference to Control	Median [d] ± SD	% Difference to Control	*p*-Value vs. Control
wild type	DMSO	18.1 ± 0.319		18.0 ± 0.340		
	PME 10 µg/mL	18.4 ± 0.341	+1.5	18.0 ± 0.387	±0	0.308
	PME 100 µg/mL	19.0 ± 0.308	+4.8	18.0 ± 0.457	±0	0.067
	PME 1000 µg/mL	21.5 ± 0.348	+18.6	22.0 ± 0.513	+22.2	<0.0001
CF1038 (mu86) [∆*daf-16*]	DMSO	10.4 ± 0.199		10.0 ± 0.199		
	PME 1000 µg/mL	10.7 ± 0.214	+3.7	10.0 ± 0.195	±0	0.073
VC199 (ok434) [∆*sir-2.1*]	DMSO	14.2 ± 0.312		14.0 ± 0.444		
	PME 1000 µg/mL	14.0 ± 0.318	−0.8	14.0 ± 0.521	±0	0.478

**Table 2:** Data of paraquat stress experiments.

Genotype	Treatment	Mean [d] ± SD	% Difference to Control	Median [d] ± SD	% Difference to Control	*p*-Value vs. Control
wild type	DMSO	68.8 ± 1.246		72.0 ± 1.212		
	PME 1000 µg/mL	74.8 ± 1.269	+8.8	72.0 ± 1.419	±0	0.0004
wild type	DMSO	71.8 ± 1.021		72.0 ± 1.084		
	PME 1 µg/mL	72.4 ± 1.192	+0.9	72.0 ± 1.274	±0	0.373
	PME 5 µg/mL	70.2 ± 1.198	−2.2	72.0 ± 1.219	±0	0.405
	PME 10 µg/mL	72.2 ± 1.139	+0.6	72.0 ± 1.170	±0	0.461
	PME 50 µg/mL	71.7 ± 1.144	±0.0	72.0 ± 1.244	±0	0.630
	PME 100 µg/mL	73.0 ± 1.140	+1.8	72.0 ± 1.174	±0	0.401
	PME 500 µg/mL	80.8 ± 0.994	+12.6	72.0 ± 1.568	±0	<0.0001
CF1038 (mu86) [∆*daf-16*]	DMSO	58.9 ± 1.184		48.0 ± 1.970		
	PME 1000 µg/mL	74.1 ± 1.419	+25.8	72.0 ± 2.020	±50.0	<0.0001
VC199 (ok434) [∆*sir-2.1*]	DMSO	87.3 ± 1.215		96.0 ± 2.168		
	PME 1000 µg/mL	84.2 ± 1.499	−3.5	96.0 ± 1.812	±0	0.053

## Supplementary Materials

Please add Supplementary Materials: The following are available online at http://www.mdpi.com/2223-7747/7/3/60/s1, Figure S1: (**A**) Effect of PME on pharingeal pumping: Wild type L4 larvae (N2) were treated for 96 h with PME or DMSO (vehicle, 0.4%) at 20 °C, then nematodes were transferred onto NGM agar-plates. Pharyngeal pumping was counted three times per nematode. Values are mean ± SD, *n* = 3 (8 individuals per group), unpaired *t*-test. (**B**) Effect of PME on offspring production: Wild type L4 larvae were treated with PME or DMSO (vehicle, 0.4%). Throughout 6 days the nematodes were transferred into new media every day and progeny was counted. Values are mean ± SD, *n* = 3 (10 individuals per group) unpaired *t*-test. (**C**) Quenching effects of PME: Fluorescent (oxidized) DCF was diluted with M9 and mixed with different concentrations of PME. The fluorescence was measured at 535 nm. Values are mean ± SD, *n* = 3 (measured in triplicates), unpaired *t*-test. (**D**) Effect of PME on the size of the nematode: Wild type L4 larvae (N2) were treated at 20 °C with PME or DMSO (vehicle, 0.4%) for 72 h, then transferred in media without PME for 24 h. Photos were taken and the size was measured by bordering the nematodes. Values are mean ± SD, *n* = 3 (20 individuals per group), one-way ANOVA with Dunnett’s multiple comparisons test., Figure S2: (**A**) Antioxidative effects (lower concentrations of PME): Wild type L4 larvae (N2) were treated with PME or DMSO (vehicle, 0.4%) for 48 h and transferred into wells containing H_2_DCF-DA. The thermally-induced increase in ROS was detected via fluorescence intensity of DCF (535 nm). Values are mean ± SD, normalized to DMSO at 420 min, *n* = 3 (16 individuals per group), one-way ANOVA with Tukey’s multiple comparisons test; (**B**) Resistance against paraquat (lower concentrations of PME): Wild type L4 larvae (N2) were treated with PME or DMSO (vehicle, 0.4%) for 72 h, then nematodes were transferred into PME-free medium containing 50 mm paraquat. The survival of the nematodes was tested by touch-provoked movement. Kaplan-Meier statistics were used for the comparison of the survival curves, *n* = 3 (60 individuals per group), Log-Rank (Mantel-Cox)-test.
