# Supplementary material for: Polygonum multiflorum Extract Exerts Antioxidative Effects and Increases Life Span and Stress Resistance in the Model Organism Caenorhabditis elegans via DAF-16 and SIR-2.1
Source: Plants (Basel). 2018 Jul 20;7(3):60. doi: 10.3390/plants7030060 (PMC6160924; doi:10.3390/plants7030060)
Supplement: Supplementary file 1 [file plants-07-00060-s001.pdf]

Supplementary Material: *Polygonum multiflorum* Extract Exerts Antioxidative Effects and Increases Life Span and Stress Resistance in the Model Organism *Caenorhabditis elegans* via DAF-16 and SIR-2.1

Christina Saier †, Christian Büchter †, Karoline Koch and Wim Wätjen *

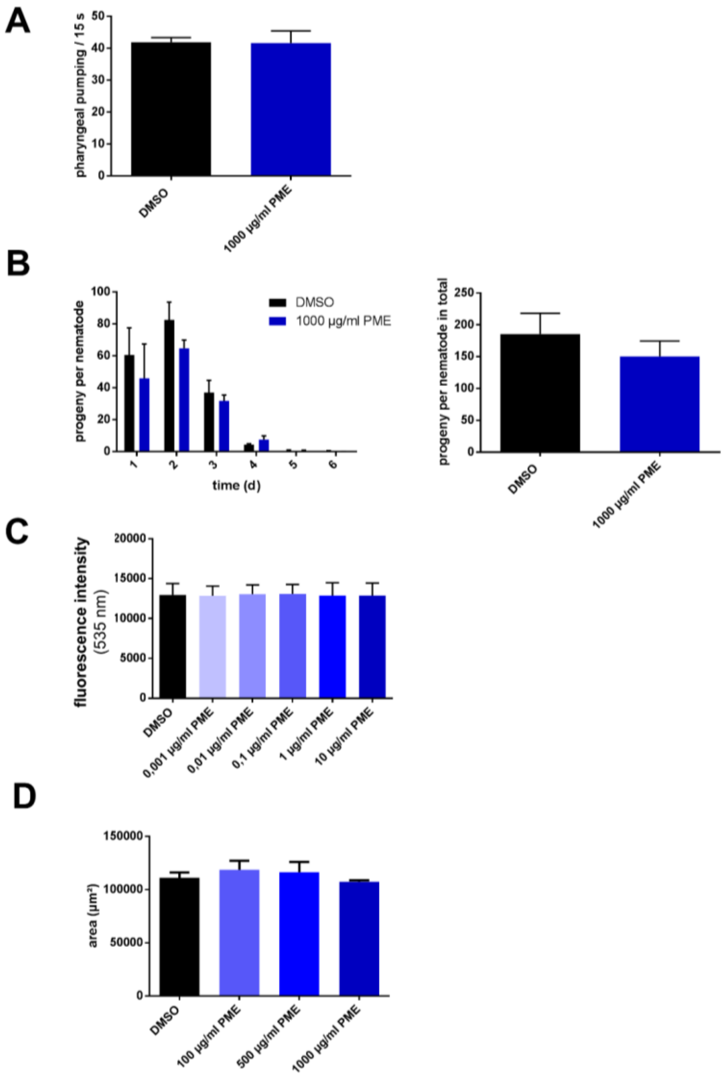

Figure S1. (**A**) Effect of PME on pharingeal pumping: Wild type L4 larvae (N2) were treated for 96 h with PME or DMSO (vehicle, 0.4%) at 20 °C, then nematodes were transferred onto NGM agar-plates. Pharyngeal pumping was counted three times per nematode. Values are mean ± SD, *n* = 3 (8 individuals per group), unpaired *t*-test. (**B**) Effect of PME on offspring production: Wild type L4 larvae were treated with PME or DMSO (vehicle, 0.4%). Throughout 6 days the nematodes were transferred into new media every day and progeny was counted. Values are mean ± SD, *n* = 3 (10 individuals per group) unpaired *t*-test. (**C**).Quenching effects of PME: Fluorescent (oxidized) DCF was diluted with M9 and mixed with different concentrations of PME. The fluorescence was measured at 535 nm. Values are mean ± SD, *n* = 3 (measured in triplicates), unpaired *t*-test. (**D**) Effect of PME on the size of the nematode: Wild type L4 larvae (N2) were treated at 20 °C with PME or DMSO (vehicle, 0.4%) for 72 h, then transferred in media without PME for 24 h. Photos were taken and the size was measured by bordering the nematodes. Values are mean ± SD, *n* = 3 (20 individuals per group), one-way ANOVA with Dunnett's multiple comparisons test.

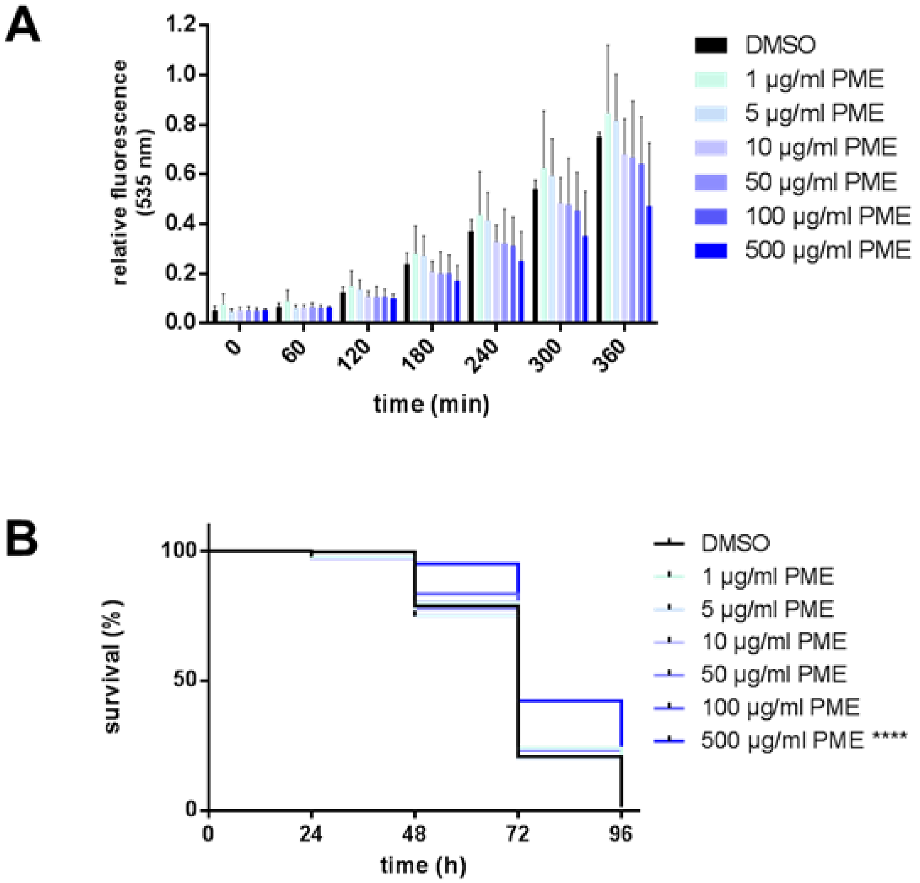

Figure S2. (**A**) Antioxidative effects (lower concentrations of PME): Wild type L4 larvae (N2) were treated with PME or DMSO (vehicle, 0.4%) for 48 h and transferred into wells containing H_2DCF-DA. The thermally-induced increase in ROS was detected via fluorescence intensity of DCF (535 nm). Values are mean ± SD, normalized to DMSO at 420 min, n = 3 (16 individuals per group), one-way ANOVA with Tukey's multiple comparisons test; (**B**) Resistance against paraquat (lower concentrations of PME): Wild type L4 larvae (N2) were treated with PME or DMSO (vehicle, 0.4%) for 72 h, then nematodes were transferred into PME-free medium containing 50 mm paraquat. The survival of the nematodes was tested by touch-provoked movement. Kaplan-Meier statistics were used for the comparison of the survival curves, n = 3 (60 individuals per group), Log-Rank (Mantel-Cox)-test.
